# Prevalence of autoantibodies against cellular antigens in patients with HIV and leprosy coinfection in the Amazon region

**DOI:** 10.1186/s40249-017-0294-2

**Published:** 2017-06-01

**Authors:** Clea Nazaré Carneiro Bichara, Carlos David Araújo Bichara, Camila Tostes, Marinete Marins Povoa, Juarez Antonio Simões Quaresma, Marília Brasil Xavier

**Affiliations:** 1 Center of Biological and Health Sciences, State University of Para, Campus II – CCBS, Rua Perebebui n° 2623, Marco, Belem, PA 66087-670 Brazil; 2 Evandro Chagas Institute, Ministry of Health, Ananindeua, PA Brazil; 3 Tropical Medicine Center, Federal University of Para, Belem, PA Brazil

**Keywords:** Leprosy, HIV, Autoantibodies, Immunology, Amazon region

## Abstract

**Background:**

Infectious agents can activate self-reactive T cells. In general, infections trigger various mechanisms, including a lack of auto-tolerance, induction of costimulatory molecules on antigen presenting cells, and molecular simulation, in addition to cross-reactions between microbial antigens and self-antigens. HIV and leprosy coinfections lead to self-immunity with the production of autoantibodies. However, not enough data on the immune behaviour associated with this coinfection are available. Therefore, this study focused on the detection of autoantibodies against cellular antigens (AACA) in individuals with HIV and leprosy coinfection in the Amazon region.

**Methods:**

Patients were distributed into four groups according to their infections: (i) coinfection with HIV and leprosy (*n* = 23), (ii) infection with leprosy (*n* = 33), (iii) infection with HIV/AIDS (*n* = 25), and (iv) healthy blood donor controls (*n* = 100). AACA were identified by indirect immunofluorescence and the samples were tested using a commercial diagnosis kit containing the antinuclear antibody HEp-2.

**Results:**

Morphologically, all stages of cell division were assessed in addition to the morphological features associated with the nuclear matrix, nucleolus, mitotic spindle, and cytoplasm. There was a high prevalence of AACA in the coinfection group (47.8%, *n* = 11) when compared with the control group of healthy blood donors (2.0%). The results showed predominantly cytoplasmic staining in all groups analysed, and no difference was observed between the presence or absence of AACA and the leprosy forms (paucibacillary and multibacillary) in the coinfection group.

**Conclusions:**

The results of this study show that despite the tendency of coinfected patients to have higher levels of autoantibodies, no correlation was observed between clinical and laboratorial variables and morbidity associated with HIV and leprosy coinfections or the levels of AACA in the serum of coinfected patients. These data are important to elucidate this complex relationship between HIV and leprosy and thus improve the follow-up of these patients.

**Electronic supplementary material:**

The online version of this article (doi:10.1186/s40249-017-0294-2) contains supplementary material, which is available to authorized users.

## Multilingual abstracts

Please see Additional file 1 for translations of the abstract into the five official working languages of the United Nations.

## Background

According to the World Health Organization (WHO), leprosy is a skin and peripheral nervous system infection cause by *Mycobacterium leprae* that persists as a public health threat. Countries such as India, Brazil, Congo, Nepal, and Mozambique are where the majority of cases are detected. In these leprosy-endemic countries, the number of cases of human immunodeficiency virus (HIV) infection is also rising [1].

From the onset of the acquired immunodeficiency syndrome (AIDS) epidemic, the possibility of coinfection in endemic areas where leprosy and HIV could occur simultaneously became a concern. So far, there is scarce information on the impact of cellular immunodeficiency on leprosy infection [2]. However, all clinical forms of leprosy have been described in AIDS patients, which suggest that the generalized immunosuppression associated with AIDS does not interfere with the clinical spectrum of leprosy [3].

The main role of the immune system is to confer organic protection through specific mechanisms that recognize foreign antigens. In certain circumstances, these mechanisms can fail and an abnormal response to host components can occur due to a lack of correct immune tolerance, the mechanism allowing antigens from the host to avoid recognition. This deregulation leads to the production of self-reactive T lymphocytes and eventually to tissue damage [4].

In some cases, depending on the pathogen-host relationship, infectious agents can activate self-reactive T cells. These infections trigger various mechanisms, including a lack of auto-tolerance, induction of costimulatory molecules on antigen presenting cells, and molecular simulation, in addition to cross-reactions between microbial antigens and self-antigens. Several studies have shown that this interaction between infectious agents and the host are not related to the presence or absence of the microorganism, but to the type of atypical immune response towards the infection [5]. Amongst the most studied infections with these responses types are viral hepatitis, mononucleosis, HIV infection, malaria, and leprosy [5–7].

Moreover, in the presence of particular stimuli, some individuals produce antinuclear factors (ANFs) that comprise a very heterogeneous group of autoantibodies, reflecting a multitude of antigens. This can be explained, in part, by the histocompatibility antigens (human leucocytes antigen, [HLA]-B8, HLA-DR2, and HLA-DQ3) present, in addition to familial genetic determinants. Production of ANFs can be a consequence of other stimuli, such as drugs and viruses, which also depend on familial determinants and histocompatibility antigens, amongst other unknown factors [8, 9].

Following exposure to elements, both nuclear and cytoplasmic, predisposed immunocompetent cells initiate antibody production against specific nuclear and cytoplasmic proteins. When in circulation, these autoantibodies can deposit in a variety of tissues and organs, thus leading to the fixation of the complement system and, as a consequence, triggering inflammation and target organ dysfunction [8–12].

The formation of the ANF complex could be an important specific marker of a series of conditions and could also contribute to their prognosis. However, the complex could also be almost irrelevant in the detection of autoimmune diseases. For this reason, its detection must always be characterized according to the fluorescence site, the patterns of deposition, the maximum dilution observed, and the clinical context [9, 11–15].

Both HIV infection and leprosy lead to self-immunity with the production of autoantibodies. However, not enough data on the immune behaviour associated with this coinfection are available. Therefore, this study focused on the detection of AACA in individuals with a HIV and leprosy coinfection in the Amazon region.

## Methods

### Study design

A transversal study in the Tropical Medicine Department of the Federal University of Pará (NMT-UFPA) was performed. In this study, the patients analysed were part of the Xavier series of patients [16] and were distributed into four groups according to their infections: (i) coinfection with HIV and leprosy (*n* = 23), (ii) infection with leprosy (*n* = 33), (iii) infection with HIV/AIDS (*n* = 25), and (iv) healthy blood donors (*n* = 100).

### Study subjects

The information was obtained at the time of leprosy diagnosis for all selected individuals, using as a reference the population of HIV-positive individuals in the state of Pará [17].

The population studied was established by means of convenience sampling and included 23 to 33 individuals per group who were admitted to the NMT-UFPA between 2000 and 2008, as well as 100 healthy blood donors sourced from the hemocenter of Pará.

For the group of patients coinfected with HIV and leprosy, the individuals considered were all HIV-positive with positive detection by serological tests (enzyme-linked immunosorbent assay, ELISA), confirmation tests using indirect immunofluorescence (IFI), and western blot. The patients were also submitted to neuro-dermatological exams and had signs and symptoms of leprosy according to the diagnosis criteria by Ridley and Jopling [18] and in agreement with the recommendations of the Ministry of Health [19], complemented by detection of acid-alcohol resistant bacilli (AARB) in the lymphs, as well as lesion histopathology.

For research purposes, epidemiological data were also collected in a data protocol file, which included the number of skin lesions, affected nerves, presence and type of reactions, development of neuritis, degree of incapacity, clinical form, stage of HIV infection, CD4+ lymphocyte count, viral burden, and comorbidity.

The viral burden and the number of CD4+ T lymphocytes were acquired by the assistant physician in the reference medical units, and the tests were performed in the Central Laboratory of the State of Pará (Laboratório Central, LACEN) of the Executive Health Office of the State of Pará (Secretaria Executiva de Saúde do Estado do Pará). CD4+ and CD8+ cell numbers were determined by flow cytometry and viral load was determined by quantitative real-time polymerase chain reaction.

Clinical and laboratorial criteria, established by the Brazilian Ministry of Health, were used to form a group of 33 leprosy-infected individuals. The absence of HIV infection was confirmed by laboratory rapid test for HIV.

Clinical and laboratorial criteria, established by the Brazilian Ministry of Health, were used to form a group of 25 HIV/AIDS-positive individuals, all of whom had no signs or symptoms of leprosy and were medically assisted by dermatologists and infectious disease physicians.

Samples of 100 blood donors who were negative for HIV and leprosy were used to form the control group.

This project was approved by the Human Research Ethics Committee of the NMT-UFPA. The participants signed an informed consent agreement. The study complied with the principles of research involving human beings (Resolution CNS 196/96-Brazil) of the National Health Board (Conselho Nacional de Saude/Brazil).

### Laboratory methods

#### Sample collection

Samples of 10 mL of blood were obtained by vacuum collection into two tubes without anticoagulant in the NMT laboratory and were stored at -20 °C until thawed for autoantibody detection and other tests.

#### Leprosy diagnosis

Leprosy diagnosis was performed according to the clinical criteria defined by the WHO and the Brazilian Ministry of Health. This was complemented by an AARB test on lymphs from four sites and included histopathology of a skin lesion obtained by biopsy.

#### HIV/AIDS diagnosis

The research was conducted according to the protocols established by the Brazilian Ministry of Health [20] and was performed in a specialized health network.

#### Detection of ANFs in HEp-2 cells

AACA detection was performed by IFI. The identification was based on the ligation of these autoantibodies to the substrate antigens. That antigen-antibody reaction was developed by the addition of a serum containing fluorescein-conjugated anti-immunoglobulin G activity.

The samples were tested using the commercial kit containing an antinuclear antibody against HEp-2 from Hemagen Diagnostics, lot 3879, with validity up to the 11th of September 2008 and the registry number 10280220029 at the Ministry of Health.

In the initial reaction steps, sera were diluted 1:40 and incubated with the substrate for 20 min, followed by washing with a buffered saline solution for 10 min. Then, the fluorescent compound was added at a 1:30 dilution and incubated for 20 min, washed for 10 min, and mounted on a buffered glycerin slide. Both positive and negative controls were included in each slide.

Slides were observed on a trinocular Leica DMLB microscope (Leica/Germany) that has a six-objective lens revolver and N plan achromatic objective lenses with infinite correction, a universal condenser NA1.30 for phase contrast as well as dark and light fields, a transmission light for light field of 100 watts to a fluorescence system, a high-pressure mercury vapour lamp of 100 watts, and conjugated fluorescein isothiocyanate filters for acridine orange, RG N2.1 for phycoerythrin, and BG 38 for fluorescein isothiocyanate. A D200 Leica digital camera (Leica/Germany) with a resolution of 2.64 megapixels, 2/3-inch CCD, and integration time of 18 images per second was annexed to the microscope for image analysis.

The morphological criteria observed were: nuclear matrix, the nucleolus, all stages of cell division, the mitotic spindle, and the cytoplasm.

### Statistical analysis

Data were stored in Microsoft Excel 2007 spreadsheets and later subjected to descriptive and analytical studies using the software packages EPI-INFO and BioEstat 5.0 (Microsoft/USA) [21], by performing the chi-square test, G-test, and Fisher’s exact test. The results were presented as frequency tables, graphs, and tables, with the established level of significance being 5% (*P* < 0.05).

## Results

### Cases

There were more men than women in all groups: in the coinfection with HIV and leprosy group, 82.6% were men; in the leprosy group, 57.6% were men; in the HIV/AIDS group, 60% were men; and out of the blood donor controls, 78% were men. Most individuals were between 21 and 40 years of age and were from Pará.

According to the WHO classification, in the coinfection group, the paucibacillary form was predominant (60.9%; *n* = 14), whereas in the leprosy only group, the multibacillary form was predominant (75.8%; *n* = 25). With respect to the immunodeficiency state, both in the coinfected and the HIV/AIDS groups, AIDS was predominant with 65.2% (*n* = 15) and 80.0% (*n* = 20), respectively (see Table 1).

**Table 1:** Distribution of patients according to the clinical form of leprosy (WHO classification) and immunodeficiency state

Groups						
Data	Group I Co-infected		Group II Leprosy		Group III HIV/AIDS	
Clinical form	*N*	%	*N*	%	*N*	%
Paucibacillary^a^	14	60.9	8	24.2	N/A	
Multibacillary^b^	9	39.1	25	75.8		
Total	23	100	33	100		
Immunodeficiency state						
AIDS	15	65.2	N/A		20	80
Not AIDS	8	34.8			5	20
Total	23	100			25	100

^a^Paucibacillary: II, BT, TT

^b^Multibacillary : BB, BL, LL

According to the Ridley and Jopling [18] classification, in the coinfected group, the borderline tuberculoid (BT) form was most prevalent (39.1%), followed by the borderline borderline (BB; 30.4%) and tuberculoid tuberculoid (TT; 21.7%) forms. In the leprosy infection group, the lepromatous form (30.3%) was the predominant form, followed by the borderline lepromatous (BL; 24.2%) and BB (21.2%) forms (see Table 2).

**Table 2:** Distribution of patients according to the clinical form of leprosy, according to the classification of Ridley and Jopling

Groups				
Data	Group I Co-infected		Group II Leprosy	
Clinical form	*N*	%	*N*	%
Tuberculoid -Tuberculoid (TT)	5	21.7	3	9.1
Boderline -Tuberculoid (BT)	9	39.1	5	15.2
Boderline - Boderline (BB)	7	30.4	7	21.2
Boderline - Lepromatous (BL)	1	4.3	8	24.2
Lepromatous-Lepromatous (LL)	1	4.3	10	30.3
Total	23	100	33	100

There was a high prevalence of AACA (*P* < 0.0001) in the coinfection group (47.8%, *n* = 11) when compared with the control group of healthy blood donors (2.0%, *n* = 2). Similar observations were made when the other groups were compared with the control group. However, no difference was observed in the prevalence of AACA between the three groups with infection (i, ii, and iii), with positive indices of 47.8%, 33.3%, and 32.0%, respectively (*P* > 0.05).

### Identification of AACA pattern

**Fig. 1:**
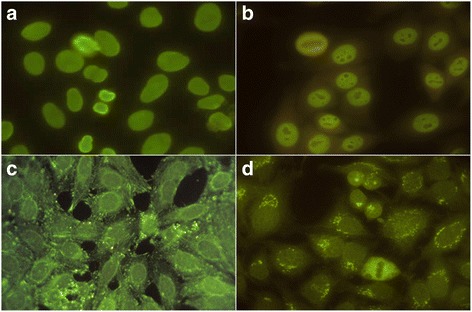
IFI on HEP-2 cells against cell antigens. **a** homogeneous nuclear pattern, **b** nuclear fine speckled, **c** linear fibrillar cytoplasmic pattern, **d** fibrillar cytoplasmic filamentary pattern

Cytoplasmic staining was predominant in all groups, including the group of healthy blood donors, with no statistically significant differences (*P* > 0.05), and the staining was of the linear fibrillar and filamentous fibrillar type. Homogenous nuclei and fine punctate nuclei were only observed in the coinfection group (27.3%) and leprosy group (9.1%), with both groups including leprosy-infected patients (see Table 3 and Fig. 1).

**Table 3:** Distribution patterns of AACA according to the study group

Autoantibody	Groups							
	Group I Co-infected		Group II Leprosy		Group III HIV/AIDS		Group IV Control	
	*N*	%	*N*	%	*N*	%	*N*	%
Nuclear	3	27.3	1	9.1	0	0	0	0
Cytoplasmic	8	72.7	10	90.9	8	100	2	100
Total	11	100	11	100	8	100	2	100

G Test (*P* > 0.2599)

### Presence of AACA in coinfected patients according to the clinical status and degree of immunodeficiency

There was no difference in the presence or absence of AACA in coinfected patients with either the paucibacillary or multibacillary forms (*P* > 0.05). However, there was a positive correlation (*P* < 0.05) for AACA positivity (*n* = 5, 55.6%) with the multibacillary form of leprosy and AIDS status, which was not observed for the paucibacillary form (*n* = 2, 14.3%; *P* > 0.05; see Table 4).

**Table 4:** Distribution of AACA in coinfected patients, according to the clinical form of leprosy and immunodeficient state

AACA and immunodeficiency state	Clinical form			
	Paucibacillary		Multibacillary	
AIDS	*N*	%	*N*	%
AACA +	2	14.3	5	55.6
AACA -	6	42.9	2	22.2
Not AIDS				
AACA +	4	28.6	0	0.0
AACA -	2	14.3	2	22.2
Total	14	100	9	100

G Test (*P* = 0.0488)

### Presence of AACA in coinfected patients and reactive status

In the coinfection group with AACA (*n* = 11), there was only one patient with a positive to leprosy reverse reaction (16.7%; *P* > 0.05; see Table 5).

**Table 5:** Distribution of AACA in coinfected patients, according to the reaction condition

Autoantibody	Reactivity state					
	With reaction		No reaction		Total	
	*N*	%	*N*	%	*N*	%
Present	1	16.7	10	58.8	11	47.8
Absent	5	83.3	7	41.2	12	52.2
Total	6	100	17	100	23	100

G Test (*P* = 0.0652)

### Correlation between levels of CD4+ cells/mm^3^ in peripheral blood and AACA

No significant correlation (*P* > 0.05) was observed between the levels of CD4+ cells/mm^3^ in the peripheral blood and the presence of AACA.

## Discussion

The idea that infectious agents cause autoimmune diseases has received considerable attention. Nevertheless, further research is required to establish the mechanisms whereby this process occurs. A better understanding of this relation may contribute to the prevention or treatment of autoimmunity-associated phenotypes, such as the production of autoantibodies, which is triggered by these agents, both isolated and together [5, 22].

The first patient with leprosy and AIDS coinfection was reported in Holland in 1990 [23], followed by reports from other countries including Brazil in 1997 [16, 24, 25]. Even today, studies about coinfection have not provided an acceptable scientific explanation, as the characteristics of the immune response in *M. leprae*-infected patients and in HIV-infected patients are not individually completely comprehended [26].

Amongst the patients analysed in this study, there were more men in all the groups and patients were mostly between 21 and 40 years old, with a significant number of patients from the state of Pará. This is in agreement with epidemiological studies of leprosy and AIDS, which found that mostly men are affected in early adulthood, and although the overall number of new leprosy cases is declining, the state of Pará continues to show high rates of the disease [24, 25, 27]. The paucibacillary clinical form was the most frequent one observed, which is in accordance with studies conducted by Xavier [16], Andrade et al. [24], and Gomes [28], which had samples from between 23 and 33 patients. The TT and BT forms were predominant, even in the presence of AIDS, which has also been reported in literature. The clinical spectrum of leprosy infection is not believed to be significantly affected by HIV coinfection [29, 30].

The presence of AACA in infected groups, whether in patients coinfected with HIV and leprosy, or leprosy or HIV infection alone, was highly significant when compared with the control group (*P* < 0.0001). This was in agreement with previous reports [22, 31] that suggest that peptides from microbial proteins with enough structural similarities to self-peptides can activate self-reactive T cells. This activation leads to autoantibody production through a mechanism known as molecular mimetism. Inflammation could also lead to the potential expansion of activated T cells [32]. This corroborates the hypothesis that the infectious process is the trigger for autoimmunity [22, 33, 34].

Specific infection by *M. leprae* did not influence autoantibody presence in HIV/AIDS patients. Similarly, HIV/AIDS did not influence the number of autoantibodies in leprosy patients. These results suggest that both agents have high potential as inducers of autoimmunity and the mechanisms involved act separately, as previously described [33–35].

There was no significant difference observed between the group of patients coinfected with HIV and leprosy, and each of the infections alone, which contradicts the hypothesis of comorbidity acting as an inducer of autoantibody production. However, there was certainly a trend toward higher levels of autoantibodies in the coinfected group (47.8%), which could be due to the fact that there are comorbidities with the immune and genetic capacity to trigger autoimmunity [16, 33, 34], a hypothesis supported by Miller et al. [32] and Rapoport et al. [36], who believe that leprosy combined with other infectious diseases can lead to an increase in autoantibody production (this study focused on HIV infection).

The results reported here show that AACA were predominant in the cytoplasm in all groups studied, including in two cases of healthy blood donors, without significant differences (*P* > 0.05). AACA were characterized as cytoplasmic linear fibrillar and cytoplasmic filamentous fibrillar.

Until now, there has been no published results concerning the mechanisms or the clinical and immunopathogenic significance of AACA presence in the cytoplasm for patients coinfected with HIV and leprosy, which limits the comprehension of the results of this study. Nonetheless, results published by Bonfá et al. [37], in which AACA levels were compared between malaria-infected patients, leprosy-infected patients, and systemic lupus erythematosus patients, showed that in the leprosy group (6/31) there was predominantly cytoplasmic staining when compared with nuclear staining, whereas in the malaria group (26/32) there was no difference in the cellular distribution, regardless of the strain of plasmodium.

According to the Brazilian Consensus of ANF in HEp-2 cells [38], AACA with cytoplasmic filamentous fibrillar staining are associated with several inflammatory and infectious diseases, whereas the cytoplasmic filamentous linear staining can be found in autoimmune hepatitis and cirrhosis. Under those same guidelines, for those patients with conditions that can lead to autoimmunity, such as infectious diseases, future discoveries may elucidate clinical aspects associated with cytoplasmic AACA. Moreover, AACA could act as important tools for the discovery of subcellular structures, and eventually the function of these autoantigens will be unravelled. These authors believe that, in the future, new developments will provide answers to important questions such as autoantigens impact on the pathogenesis of autoimmune diseases.

A nuclear pattern of AACA was observed in coinfected patients (27.3%) and in patients with leprosy infection (9.1%), but it was absent in the HIV/AIDS group and in the control group, without statistical significance between groups (*P* > 0.05). Meanwhile, because AACA were only detected in coinfected and leprosy-infected patients, this suggests that *M. leprae* infection could induce the production of autoantibodies with nuclear distribution.

This possibility has been investigated by searching for AACA nuclear distribution in leprosy patients. Dacas et al. [35] performed a meta-analysis involving 10 reports on the prevalence of ANFs using IFI/HEp-2, and the results varied between 0% and 85.7% [39]. The high variability was attributed to differences in clinical forms, age, and genetic factors of the populations studied [36, 37]. The authors also report that AACA could be detected in healthy individuals, who are considered normal. According to their results, a nuclear pattern was observed in 16.7% of healthy individuals, whereas in the present work, the control group had no nuclear. Miller et al. [32] tested the serum of 46 patients with leprosy infection using a panel of antinuclear antibodies and detected antinuclear antibodies in 16% of cases, in agreement with a report by Rapoport et al. [36], who studied a series of autoantibodies in patients with mycobacterium infection and detected 7.3% seropositive cases amongst 41 patients with leprosy.

In contrast with the present study, which did not detect nuclear AACA in patients of the HIV/AIDS group, several reports, such as Kopelman and Zolla-Pazner [40], have previously described the presence of these autoantibodies in individuals with HIV/AIDS, presumably without leprosy infection.

In the group with HIV and leprosy coinfection, the presence or absence of AACA did not correlate with the multibacillary or paucibacillary forms (*P* > 0.05). It should be highlighted that, in this work, the paucibacillary forms were predominant and amongst the multibacillary group, there was only one patient with the polar lepromatous form, whereas all the others had borderline forms. As such, the non-bacillary forms may have influenced the results. There is, however, controversy on this subject, with the majority of authors favouring the hypothesis that there is no difference between the various forms of leprosy. Dacas et al. [35] studied 120 leprosy patients in southern Brazil (77 multibacillary and 43 paucibacillary) and observed that 55.8% of the patients were ANF positive (*P* < 0.0001), with similar distributions between the paucibacillary and multibacillary forms, though with higher prevalence in the multibacillary group.

The significant prevalence of AACA in multibacillary patients with AIDS could allude to an increased humoral response to *M. leprae,* linked to HIV infection. The cell death could induce immune deregulation during infection and be associated with a shift towards a cytokine profile compatible with Th2 type humoral responses [34, 41].

The presence of reactivity associated with leprosy did not influence the presence of AACA in the coinfection group (*P* > 0.05). These results are in agreement with Edington et al. [42], who searched for nuclear AACA in 20 patients not coinfected and with reactivity, and did not detect AACA. However, the small number of patients with reactivity among the coinfected individuals in this study does not agree with data from two of Brazil’s largest studies conducted by Xavier [16], with 31 patients, and Gomes [28], with 30, which observed high numbers of reactive episodes in their patients.

The levels of CD4+ T cells in the peripheral blood of the 23 coinfected patients were, on average, 245.44 cells/mm^3^ for patients with AACA and 293 cells/mm^3^ for patients negative for AACA. The lack of statistical significance (*P* > 0.05) shows that the autoantibody production was independent from the levels of CD4+ peripheral T cells. These results could be compared with the results of Xavier [16], who counted over 200 cells/mL of CD4+ T cells in the majority of the 31 patients analysed. This result was independent of the clinical forms of leprosy, the presence of *M. leprae* specific antibodies, and the reactivity status. Similarly, Gomes [28] observed that in 20 patients with coinfection, the levels of CD4+ T cells varied between undetectable and 1253 cells/mm^3^, without correlation with other variables.

Finally, the relatively small sample size of patients coinfected limited the statistical analysis. The sample size is small due to difficulties in obtaining patients who have HIV and leprosy coinfection. These issues point to the need for further studies to better characterize the behaviour of autoimmunity mechanisms. In particular, studies relating to the production of autoantibodies and the blood cytokine and immunogenetic profiles could contribute answers to the gaps in knowledge about this relationship and open up new areas of scientific research.

## Conclusions

The results reported here suggest that despite coinfected patients having a higher tendency to produce autoantibodies, the effects of the clinical and laboratorial variables were weak for morbidity associated with HIV and leprosy coinfection, as well as for the production of AACA in the serum of those patients. The relatively small sample of patients limited the results. As such, variables that can modulate these processes should be investigated using larger patient cohorts in order to identify clinical and immunological responses for the results obtained.

## Additional file

Additional file 1: Multilingual abstracts in the five official working languages of the United Nations. (PDF 544 kb)

## Abbreviations

AACA: Autoantibodies against cellular antigens
AARB: Acid-alcohol resistant bacilli
AIDS: Acquired immunodeficiency syndrome
ANF: Antinuclear factor
BB: Borderline borderline
BT: Borderline tuberculoid
HIV: Human immunodeficiency virus
HLA: Human leucocytes antigen
IFI: Indirect immunofluorescence
LACEN: Laboratório Central
NMT: Núcleo de Medicina Tropical (Tropical Medicine Department)
TT: Tuberculoid tuberculoid
UFPA: Universidade Federal do Pará (Federal University of Pará)
WHO: World Health Organization
