# Supplementary material for: Prevalence of autoantibodies against cellular antigens in patients with HIV and leprosy coinfection in the Amazon region
Source: Infect Dis Poverty. 2017 Jun 1;6:80. doi: 10.1186/s40249-017-0294-2 (PMC5452639; doi:10.1186/s40249-017-0294-2)
Supplement: Additional file 1: — Multilingual abstracts in the five official working languages of the United Nations. (PDF 544 kb) [file 40249_2017_294_MOESM1_ESM.pdf]

Translation of the abstract into the five official working languages of the United Nations

انتشار الأجسام المضادة ضد المستضدات الخلوية في المرضى الذين يعانون من فيروس نقص المناعة البشرية المصحوبة بعدوى الجذام في منطقة الأمازون

كليا نازاري كارنيرو بشارة، كارلوس ديفيد أروجو بشارة، كاميلا توتيس، مارنيتي مارينز بوفوا، خواريز انطونيو سيمويس كواريسما، ماريليا البرازيل كزافييه

ملخص

خلفية: يمكن للعوامل المسببة للعدوى أن تنشط ا الخلايا الليمفاوية ت ذاتية التفاعل. بشكل عام، تؤدي الالتهابات لبدء عمل آليات مختلفة، بما في ذلك عدم وجود التسامح المناعي الذاتي وحث جزيئات المفعل المشارك على الخلايا المقدمة للمستضد، والمحاكاة الجزيئية، بالإضافة إلى ردود الفعل المتبادلة بين المضادات الميكروبية والمستضدات الذاتية. فيروس نقص المناعة البشرية المصحوبة بعدوى الجذام تؤدي إلى مناعة ذاتية مع إنتاج الأجسام المضادة. ومع ذلك، لا تتوفر بيانات كافية على السلوك المناعي المرتبط بهذه العدوى المصاحبة. لذلك، ركزت هذه الدراسة على الكشف عن الأجسام المضادة ضد المستضدات الخلوية في الأفراد الذين يعانون من فيروس نقص المناعة البشرية المصحوبة بعدوى الجذام في منطقة الأمازون.
الأساليب: تم توزيع المرضى إلى أربع مجموعات وفقا للعدوى. (أ) عدوى فيروس نقص المناعة البشرية مصحوبة بمرض الجذام (n = 23)، (ب) عدوى الجذام (n = 33)، (ج) الإصابة بفيروس نقص المناعة البشرية / الإيدز (n = 25)، (د) ضوابط التبرع بالدم صحي (n = 100). وقد تم تحديد الأجسام المضادة ضد المستضدات الخلوية بالفلورة المناعية غير المباشرة وتم اختبار العينات باستخدام مجموعة تشخيص التجارية التي تحتوي على الأجسام المضادة للنواة HEp-2.
النتائج: شكليا، تم تقييم جميع مراحل الانقسام الخلوي بالإضافة إلى الميزات المورفولوجية المرتبطة بالمصفوفة النووية، النوية، مِغْزَلُ الانْقِسام التَّفَتُّلِيّ، السيتوبلازم. كان هناك ارتفاع في معدل انتشار الأجسام المضادة ضد المستضدات الخلوية في مجموعة العدوى المصاحبة (47.8٪، n = 11) مقارنة مع مجموعة التحكم من المتبرعين بالدم (2.0%). أظهرت النتائج تَلَوُّن هَيولِيّ بشكل مسيطر في جميع الفئات التي تم تحليلها، ولوحظ عدم وجود فرق بين وجود أو عدم وجود الأجسام المضادة ضد المستضدات الخلوية وأشكال الجذام (قليل العصيات ومتعدد العصيات) في المجموعة المصحوبة بالعدوى.
الاستنتاجات: تظهر نتائج هذه الدراسة أنه على الرغم من ميل المرضى المصابون بعدوى مصاحبة بمستويات أعلى من الأجسام المضادة، لوحظ عدم وجود ارتباط بين المتغيرات السريرية والمخبرية والأمراض المرتبطة بفيروس نقص المناعة البشرية المصحوبة بعدوى الجذام أو مستويات الأجسام المضادة ضد المستضدات الخلوية في مصل المرضى المصابين بعدوى مصاحبة. وهذه البيانات مهمة لتوضيح هذه العلاقة المعقدة بين فيروس نقص المناعة البشرية ومرض الجذام، وبالتالي تحسين متابعة هؤلاء المرضى.

Translated from English version into Arabic by Mahmoud Sami, through

TRANSLATORS
WITHOUT BORDERS

亚马逊地区 **HIV** 和麻风合并感染患者抗细胞抗原自身抗体流行情况

Clea Nazaré Carneiro Bichara, Carlos David Araújo Bichara, Camila Tostes, Marinete Marins Povoa, Juarez Antonio Simões Quaresma, Marília Brasil Xavier

摘要

引言：传染病原可以激活自身反应性 T 细胞。通常，感染触发多种机制，包括自身耐受的缺乏、诱导抗原提呈细胞表达共刺激因子、以及分子模拟，除此之外，还有微生物抗原与自身抗原之间的交叉反应。然而，并没有足够的与合并感染相关的免疫行为数据可供参考。因此，本研究致力于检测亚马逊地区 HIV 和麻风合并感染患者的抗细胞抗原自身抗体（AACA）的表达情况。
方法：根据患者所患传染病情况，将其分为四组：(i) 合并感染 HIV 和麻风组(*n* = 23), (ii)感染麻风组(*n* = 33), (iii) 感染 HIV/AIDS 组(*n* = 25), (iv)健康献血对照组(*n* = 100)。通过间接免疫荧光检测抗细胞抗原自身抗体，并使用含抗核抗体 HEp-2 的商业诊断试剂盒对样本进行检测。
结果：形态学上，除了评估与形态学特征相关的核基质、核仁、有丝分裂纺锤体以及胞浆之外，还对细胞分裂的所有阶段进行评估。与对照组健康献血者相比(2.0%)，合并感染组 AACA 的发生率较高 (47.8%, *n* = 11)。结果显示，所有组的胞浆均着色明显；在合并感染组，是否存在 AACA，不同麻风类型（少菌型和多菌型）之间未见差异。
结论：本研究表明，尽管合并感染患者具有较高抗细胞抗原自身抗体水平，然而临床和实验室变量与合并感染 HIV 和麻风的发病率或者合并感染患者血清中 AACA 水平之间无关联，这些数据对于阐明 HIV 和麻风之间的复杂关系是十分重要的，进而可改善这些患者的随访。

Translated from English version into Chinese by Lei Sun, edited by Pin Yang

CHINESE CENTER FOR DISEASE CONTROL AND PREVENTION
NATIONAL INSTITUTE OF PARASITIC DISEASES

Prédominance d'auto-anticorps contre les antigènes cellulaires chez les patients co-infectés par le VIH et la lèpre dans la région amazonienne

Clea Nazaré Carneiro Bichara, Carlos David Araújo Bichara, Camila Tostes, Marinete Marins Povoa, Juarez Antonio Simões Quaresma, Marília Brasil Xavier

RESUME
Contexte: les agents infectieux peuvent activer les cellules T auto-réactives. En général, les infections déclenchent divers mécanismes, y compris un manque d'auto-tolérance, une induction de molécules de costimulation sur les antigènes présentant des cellules, et une stimulation moléculaire, en plus des réactions croisées entre les antigènes microbiens et les auto-antigènes. Les co-infections par le VIH et la lèpre conduisent à une auto-résistance avec la production d'anticorps. Cependant, il n'y a pas suffisamment de données disponibles sur le comportement immunitaire associé à cette co-infection. Par conséquent, cette étude s'est focalisée sur la détection des auto-anticorps contre les antigènes cellulaires (AAAC) chez les personnes co-infectées par le VIH et la lèpre dans la région amazonienne.
Méthodes: les patients ont été répartis en quatre groupes en fonction de leurs infections:
(i) co-infection par le VIH et la lèpre (*n* = 23), (ii) infection par la lèpre (*n* = 33), (iii) infection par le VIH / SIDA (*n* = 25), et (iv) contrôles du sang de donneurs sains (*n* = 100). Les AAAC ont été identifiés par immunofluorescence indirecte, et les échantillons ont été analysés en utilisant un kit commercial de diagnostic contenant l'anticorps antinucléaire HEp-2.

Résultats: Morphologiquement, tous les stades de division cellulaire ont été évalués en plus des caractéristiques morphologiques associées à la matrice nucléaire, au nucléole, au fuseau mitotique, et au cytoplasme. On a noté une forte prédominance de AAAC dans le groupe co-infecté (47,8%, *n* = 11) comparé au groupe de contrôle du sang des donneurs sains (2,0%). Les résultats ont montré une coloration cytoplasmique dans tous les groupes analysés, et aucune différence n'a été observée entre la présence ou l'absence d'AAAC et les formes de lèpre (paucibacillaire et multibacillaire) dans le groupe co-infecté.
Conclusions: les résultats de cette étude montrent que malgré la tendance des patients co-infectés a avoir des niveaux d'auto-anticorps plus élevés, aucune corrélation n'a été observée entre les variables cliniques et de laboratoire, et la morbidité associée aux co-infections par le VIH et la lèpre, ou des niveaux de AAAC dans le sérum des patients co-infectés. Ces données sont importantes pour éclairer la relation complexe entre le VIH et la lèpre, et donc améliorer le suivi de ces patients.

Translated from English version into French by Ode Laforge, through

TRANSLATORS
WITHOUT BORDERS

Распространенность аутоантител против клеточных антигенов у пациентов с ВИЧ-инфекцией и проказой в Амазонии

Clea Nazaré Carneiro Bichara, Carlos David Araújo Bichara, Camila Tostes, Marinete Marins Povoa, Juarez Antonio Simões Quaresma, Marília Brasil Xavier

Реферат

Фон: Инфекционные агенты могут активировать самореактивные Т-лимфоциты. В общем, инфекции запускают различные механизмы, в том числе и отсутствие автоматической толерантности, индукция костимулирующих молекул в антиген-представляющих клетках, и молекулярном моделировании, в дополнение к перекрестной реакции между антигенами микробных и собственных антигенов. ВИЧ и лепра коинфекции приведут к самозащите с продукцией аутоантител. Однако, недостаточно данных об иммунном поведении, которые связанны с коинфекцией доступны. Таким образом, данное исследование ориентировано на выявление аутоантител против клеточных антигенов (предлагает) у лиц с ВИЧ-инфекцией и проказой в регионе Амазонки.
Методы: Пациенты были распределены на четыре группы в зависимости от их инфекций: (i) коинфекция с ВИЧ и лепра (*n*= 23), (ii) и заражения проказой (*n*= 33), (iii) и ВИЧ/СПИДа (*n*= 25) и (IV) крови здоровых доноров контролем (*n* = 100). Проекта AACA были определены непрямой иммунофлюоресценции и образцы были протестированы с использованием коммерческого диагноза комплект, содержащий антиядерные антитела HEp-2.
Результаты: Морфологически все этапы деления клетки были оценены, помимо морфологических особенностей, связанных с ядерным матриксом, ядрышко, митотическим веретеном, а в цитоплазме. Существует высокая распространенность предлагает в

коинфекция группы (47.8%, *n* = 11) по сравнению с контрольной группой здоровых доноров крови (2.0%). Результаты показали, преимущественно цитоплазматические окрашивания во всех группах проанализированы, и никакой разницы не наблюдалось между наличием или отсутствием проекта AACA и проказы форм (paucibacillary и multibacillary) в группе сочетанной инфекции.

Заключение: Результаты этого исследования показывают, что, несмотря на склонность пациентов с коинфекцией, которые имеют более высокий уровень аутоантител, не было выявлено корреляции между клинико-лабораторными переменными и заболеваемостью, которые связанны с ВИЧ и лепрой коинфекции или уровней при AACA в сыворотке крови пациентов с коинфекцией. Эти данные важны для выяснения этой сложной взаимосвязи между ВИЧ-инфекцией и проказой, и таким образом улучшить контроль за этими пациентами.

Translated from English version into Russian by Hao-Qi Zhang

Prevalencia de autoanticuerpos contra antígenos celulares en pacientes con coinfección por HIV y lepra en la región Amazónica.

Clea Nazaré Carneiro Bichara, Carlos David Araújo Bichara, Camila Tostes, Marinete Marins Povoa, Juarez Antonio Simões Quaresma, Marília Brasil Xavier

RESUMEN

Antecedentes: los agentes infecciosos pueden activar las células T auto reactivas. Generalmente, las infecciones disparan varios mecanismos que incluyen la falta de auto tolerancia, inducción de moléculas co estimuladoras en células presentadoras de antígenos y simulación molecular, además de las reacciones cruzadas entre los antígenos microbianos y antígenos propios. Las coinfecciones por HIV y lepra conducen hacia la autoinmunidad con la producción de anticuerpos. Sin embargo, no existen datos suficientes disponibles relacionados con esta coinfección. Por lo tanto, este estudio se enfoca en la detección de autoanticuerpos contra los antígenos celulares (AACA) en individuos con coinfecciones por HIV y lepra en la región Amazónica

Métodos: los pacientes se dividieron en cuatro grupos según sus infecciones: (i) coinfección por HIV y lepra (*n* = 23), (ii) infección por lepra (*n* = 33), (iii) infección por HIV/SIDA (*n* = 25), Y (iv) controles de donantes de sangre sanos (*n* = 100). Los AACA se identificaron con inmunofluorescencia indirecta y las muestras se testearon con un kit de diagnóstico comercial que contenía el anticuerpo antinuclear HEp-2.

Resultados: morfológicamente, todas las etapas de la división celular se evaluaron además de las características morfológicas relacionadas con la matriz celular, el núcleo, huso mitótico y el citoplasma. Hubo una alta prevalencia de AACA en el grupo de coinfecciones (47.8%, *n* = 11) al compararlo con el grupo de donantes e sangre saludables (2.0%). Los resultados mostraron una tinción citoplasmática predominante en todos los grupos analizados y no se observó ninguna diferencia entre la presencia o ausencia de AACA en las lepras paucibacilar y multibacilar en el grupo de coinfecciones.

Conclusiones: Los resultados de este estudio muestran que a pesar de la tendencia de los pacientes coinfectados de presentar niveles más altos de autoanticuerpos, no se observó ninguna correlación entre las variables clínicas y de laboratorio ni con la morbilidad relacionada con las coinfecciones por HIV y lepra, o los niveles de AACA en el plasma de los pacientes coinfectados. Estos datos son importantes para dilucidar esta relación compleja entre el HIV y la lepra, y por lo tanto, mejorar el seguimiento de estos pacientes.

Translated from English version into Spanish by M. Kaszczynice, through

TRANSLATORS
WITHOUT BORDERS
